# Targeting PI3K in Cancer: Any Good News?

**DOI:** 10.3389/fonc.2013.00108

**Published:** 2013-05-08

**Authors:** Miriam Martini, Elisa Ciraolo, Federico Gulluni, Emilio Hirsch

**Affiliations:** ^1^Molecular Biotechnology Center, University of TurinTurin, Italy

**Keywords:** PI3K, cancer, therapeutics, genetic determinants, class II phosphatidylinositol 3-kinase

## Abstract

The phosphatidylinositol 3-kinase (PI3K) signaling pathway regulates several cellular processes and it’s one of the most frequently deregulated pathway in human tumors. Given its prominent role in cancer, there is great interest in the development of inhibitors able to target several members of PI3K signaling pathway in clinical trials. These drug candidates include PI3K inhibitors, both pan- and isoform-specific inhibitors, AKT, mTOR, and dual PI3K/mTOR inhibitors. As novel compounds progress into clinical trials, it’s becoming urgent to identify and select patient population that most likely benefit from PI3K inhibition. In this review we will discuss individual *PIK3CA* mutations as predictors of sensitivity and resistance to targeted therapies, leading to use of novel PI3K/mTOR/AKT inhibitors to a more “personalized” treatment.

## Introduction

Over the past years, it has become widely accepted that cancer is a multistep genetic disease that arises by the activation of specific oncogenes, inactivation of tumor suppressor genes, and stochastic accumulation of genetic alterations driving tumor progression (Vogelstein and Kinzler, 2004). Despite the genetic and epigenetic complexity observed in cancer, tumor growth and survival can be impaired by the inactivation of a single oncogene. This phenomenon is called “oncogene addiction” (term coined by Weinstein, 2000), and reveals a possible “Achilles heel” within the cancer cell that can be therapeutically exploited (Weinstein, 2000, 2002). The hypothesis of oncogene addiction refers to the observation that a tumor cell, despite several genetic alterations, is dependent on a single oncogenic pathway responsible for sustaining the malignant phenotype. An important implication is that switching off this crucial pathway upon which cancer cells are dependent should have negative effects on cancer while sparing normal cells. Therefore pharmacological inhibition of this crucial pathway cause an “addiction shock,” resulting in the blockade of cell growth or in cell death (Sharma and Settleman, 2007; Janne et al., 2009).

The “addiction paradigm” has been pharmacologically exploited and drugs designed to specifically inhibit mutated proteins have led to what is commonly known as “personalized cancer medicine.” At present only a small subset of anticancer therapies are administered based upon the genetic alterations present in individual tumors (Martini et al., 2012). For example, breast cancer patients with amplification/overexpression of the human epidermal growth factor receptor (EGFR) 2 (HER-2) are selectively sensitive to Trastuzumab and Lapatinib (Stern, 2012), melanomas harboring *BRAF* V600E mutations to Vemurafenib (Flaherty et al., 2010), non-small cells lung cancers (NSCLC) with mutated EGFR to Erlotinib and Gefitinib (Pallis et al., 2011), and KIT and PDGFRA mutant gastrointestinal stromal tumors (GIST) to Imatinib (Antonescu, 2011).

The phosphatidylinositol 3-kinase (PI3K) signaling pathway regulates several processes in normal cell such as survival, metabolism, and motility and it’s one of the most frequently deregulated pathway in human cancer (Cantley, 2002; Samuels et al., 2004; Liu et al., 2009a). Mutations and/or amplifications of the PI3K catalytic subunits p110α (*PIK3CA*) and p110β (*PIK3CB*), the PI3K regulatory subunits p85α (*PIK3R1*) and p85β (*PIK3R2*), the PI3K effector AKT (*AKT1*) are often observed in cancer^1^. Moreover, mutations, deletions, or epigenetic changes of negative regulators of PI3K axis (Gewinner et al., 2009; Hollander et al., 2011), such as phosphatase and tensin homolog (PTEN) and inositol polyphosphate-4-phosphatase, type II (INPP4B), may alter sensitivity to chemo- and targeted-therapies (Steelman et al., 2008; Kim et al., 2012).

While class I is the most well characterized of the PI3K to date, the family comprises other two groups; class II and class III. Each class of PI3-kinase has unique preferences for phosphoinositide substrates and produces specific lipid second messengers, responding to a wide variety of signaling molecules.

Class II PI3Ks consists of three members named PI3K-C2α, PI3K-C2β, and PI3K-C2γ. Unlike class I, they lack a regulative subunit and appear to be monomers of high molecular weight, predominantly associated with intracellular membranes (Falasca et al., 2007). PI3K-C2α and PI3K-C2β have a broad tissue distribution and are almost ubiquitously expressed, while PI3K-C2γ displayed a very restricted expression pattern, limited to liver, pancreas, and prostate (Kok et al., 2009). The precise nature of class II substrates and lipid products is still debated. Class II members are thought to act similarly to class III mainly generating PtdIns(3)P *in vitro* and *in vivo* (Falasca and Maffucci, 2007), nonetheless, they can also produce PtdIns(3,4)P_2_ *in vitro* (Vanhaesebroeck et al., 2010) and PI3K-C2α has been also reported to produce PtdIns(3,4,5)P_3_ *in vitro* (Gaidarov et al., 2001). Class II PI3Ks are activated downstream of different receptor types including RTKs (EGFR and PDGFR) (Brown et al., 1999; Arcaro et al., 2000, 2002; Falasca and Maffucci, 2007) and GPCRs (Maffucci et al., 2005). Several stimuli promote PI3K-C2α activation such as hormones (insulin) (Brown et al., 1999), chemokines (Turner et al., 1998), and cytokines (TNFα and leptin) (Ktori et al., 2003). Similarly PI3K-C2β is activated by growth factor (EGF) (Arcaro et al., 2002) and phospholipids (LPA) (Maffucci et al., 2005) while at the present there are no study investigating PI3K-C2γ upstream activators. A recent study reported that PI3K-C2α has an essential role in angiogenesis resulting in embryo lethality, impaired endothelial cell signaling, and RhoA activation (Yoshioka et al., 2012).

The increasing understanding of the mechanisms underlying the role of the PI3K pathway in tumorigenesis has encouraged many pharmaceutical companies and academic laboratories to focus their efforts on the development of inhibitors targeting the PI3K signaling pathway at different levels.

In this review, we will discuss the challenges for the development of novel inhibitors to target the PI3K signaling pathway and the binary relationship between PI3K mutations in cancer genotype and personalized medicine.

## Targeting PI3K Signaling Pathway in Cancer

**Figure 1:**
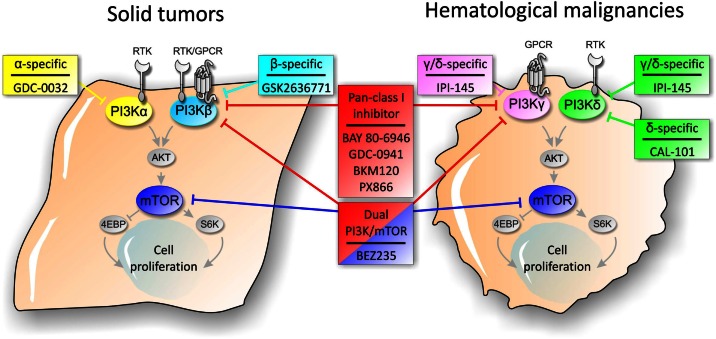
**Schematic representation of PI3K pathway and sites of action of PI3K signaling pathway inhibitors in solid tumors and hematological malignancies**.

Since PI3K/AKT/mTOR axis has been classified among the most frequently activated pathway in cancer, members of the cascade represent an attractive target for cancer therapeutics (Miled et al., 2007). The activation of the PI3K signaling pathway contributes to several aspects of tumorigenesis as tumor development, progression, invasiveness, and metastasis formation. A number of molecules targeting members of the PI3K axis have been developed and evaluated in preclinical studies as well as in clinical trials (Figure 1). Based on pharmacokinetics properties and isoform selectivity for the ATP binding site, PI3K inhibitors have been classified into different groups (Table 1).

**Table 1:** **PI3K inhibitors tested in preclinical and clinical models, genetic determinants of response, and open clinical trials described for each compound**.

Group	Selectivity	Compound	Cancer type	Genetic determinant of response	Clinical trial status
I Pan-class I	Class I PI3K	GDC-0941 (Roche/Genentech)	Breast	HER-2 amplification PIK3CA mutations	I-II in breast, non-Hodgkin’s lymphoma, NSCLC
		
			Melanomas, MM, non-Hodgkin’s lymphoma, NSCLC, ovarian	–	
	
		BKM120 (Novartis)	Breast	PIK3CA mutations	I-II in breast CRC, endometrial, GIST, GBM, leukemia, melanoma, NSCLC, pancreatic, renal cell, SCCHN, TCC
		
			CRC, endometrial, GIST, GBM, leukemia, melanoma, NSCLC, pancreatic prostate	–	
	
		PX866 (Oncothyreon)	Breast, CRC, MM, NSCLC, pancreatic prostate, ovarian	PIK3CA mutation, PTEN loss	I-II in CRC, GBM, NSCLC, SCCHN
	
		BAY 80-6946 (Bayer)	Advanced solid cancers	PIK3CA mutations	I-II in advanced solid cancers

II Isoform specific	PI3Kα	GDC-0032 (Roche/Genentech)	Solid cancers	–	I-II in solid cancers
	
	PI3Kβ	GSK2636771 (GlaxoSmithKline)	Advanced solid cancers	–	I-IIa in advanced solid cancers with PTEN deficiency
	
	PI3Kγ and PI3Kδ	IPI-145 (Infinity)	Hematological malignancies	–	I-IIa in advanced hematological malignancies
	
	PI3Kδ	CAL-101 (Gilead Sciences)	AML, CLL, Hodgkin’s and non-Hodgkin’s lymphoma, MCL	–	I-II-III in AML, CLL, Hodgkin’s and non-Hodgkin’s lymphoma, MCL, MM

III Dual PI3K/mTOR	PI3K and mTOR	BEZ235 (Novartis)	Breast	PIK3C2a mutation, HER-2 amplification, PTEN loss	I-II in breast, renal cell
		
			Ovarian	PIK3C2a mutation, PTEN loss	

The first group encompasses inhibitors able to bind all class I PI3Ks (pan inhibitors), and in particular PI3Kα, PI3Kβ, PI3Kγ, and PI3Kδ. Wortmannin and LY294002, the first two prototype PI3K inhibitors, represented for a long time a useful tool in the study of PI3K function in cellular processes, given their effectiveness at low concentration (nM). Nevertheless, given their poor pharmacokinetic properties and lack of selectivity, these compounds have limited their therapeutic potential. The availability of the crystal structure of the p110 isoform-specific catalytic subunits gave a boost to the development of new PI3K inhibitors (Vadas et al., 2011). Therefore, several novel compounds have been further developed in order to improve pharmacokinetic profiles, to increase target specificity and to minimize toxicity. At the present, several promising pan-PI3K inhibitors are under development and evaluation in clinical trials for cancer therapy. These molecules predominantly display cytostatic effects with consequent G1 phase arrest *in vitro* and favorable anticancer effects *in vivo*.

Lately, a second group of PI3K inhibitors has been developed to overcome the toxicity displayed by the treatment with pan-PI3K inhibitors. They are characterized by greater selective activity (isoform-specific) and several molecules are currently under evaluation in preclinical and clinical studies.

On the other hand, since PI3K and mTOR share several structural similarities, many chemical compounds, under evaluation in clinical trials, are able to inhibit both catalytic subunits. This third group of inhibitors is termed “dual PI3K/mTOR” and they have the advantage of inhibiting not only all class I isoforms but also mTORC1 and mTORC2 thus having a strongest effectiveness in switching off the PI3K signaling pathway.

Given the growing interest in inhibiting PI3K signaling pathway, the drug development landscape is becoming increasingly crowded and highly competitive, so that several pharmaceutical companies, such as Novartis, Sanofi-Aventis, Roche/Genentech, Bayer, and GlaxoSmithKline, are currently in competition to bring their own inhibitors in the market. On the basis of these considerations, we will describe and discuss results for the most important PI3K inhibitors currently in clinical trial.

## PI3K Signaling Inhibition in Solid Tumors

Several PI3K inhibitors have progressed through early clinical safety and dose-escalation studies to phase I clinical trials on solid tumors. This allowed to define the treatment efficacy and to identify patient population that will benefit from PI3K inhibitor administration. Furthermore, a few number of compounds are now progressing to phase Ib expansion cohort and phase II single agent efficacy studies.

Several early reports were presented in the last 3 years revealing safety and some preliminary activity data about the use of class I PI3K and dual mTOR/PI3K inhibitors in patients.

### GDC-0941 (Genentech-Roche)

GDC-0941 is a potent and selective oral inhibitor of class I PI3K with activity against DNA-PK and also mTOR but at high concentrations (Folkes et al., 2008). GDC-0941 is currently under evaluation in several phase I clinical trials on patients with advanced solid tumors, such as HER-2 positive metastatic breast cancer and advanced NSCLC^2^. Several studies have documented the effects of GDC-0941 on cell viability inhibition at submicromolar concentration in several tumor types including glioblastoma, breast, and prostate cell lines carrying specific alteration in the PI3K signaling pathway. GDC-0941 exhibited excellent inhibition on MCF7, T-47D, and SK-BR-3 breast derived cancer cell lines as a single agent while in combination with rapamycin promotes apoptosis and down regulates cell cycle machinery components, such as cyclin D1 (Zheng et al., 2012). In addition, the combinatorial treatment of GDC-0941 and the Docetaxel in a panel of 25 breast tumor cell lines (HER-2+, luminal, and basal subtypes) increases the rate of apoptosis and enhances sensitivity to Docetaxel (Wallin et al., 2012). The efficacy of combined GDC-0941 and chemo- and targeted-therapies has also been demonstrated for other agents such as Trastuzumab, Pertuzumab, and Docetaxel (Yao et al., 2009). In NSCLC GDC-0941 synergizes also with the MEK inhibitor (U0126) by promoting G0-G1 arrest and cell apoptosis (Zou et al., 2012). Several clinical studies are currently evaluating the relative bioavailability, absorption, metabolism, excretion, and effects of GDC-0941 in patient with advanced or metastatic tumors, alone or in combination with Paclitaxel, Erlotinib, Carboplatin, or Bevacizumab (see text footnote 2) GDC-0973 (MEK inhibitor). Safety data about GDC-0941 monotherapy regimen have been recently released. Phase I studies with a 3 + 3 dose-escalation design showed that GDC-0941 is generally well tolerated at doses below 450 mg every day (QD) and twice a day (BID) in patient with advanced solid tumors. After treatment, signs of clinical activity have been reported, including a partial response (PR) by the response evaluation criteria in solid tumors (RECIST) in a patients with melanoma, ovarian, endocervical, and ER+/HER-breast cancer (Wagner et al., 2009; Moreno Garcia et al., 2011; Von Hoff et al., 2011). Several side effects have been described after administration of GDC-0941 in about 10% of patients, in particular nausea, diarrhea, fatigue, dysgeusia, and decreased appetite. In two different studies, the maximum tolerated dose (MTD) was enriched at 450 mg with a dose limiting toxicities (DLT) of grade 3 (Gr) macular rash and asymptomatic *T*-wave inversion in ECG, Gr3 thrombocytopenia, and Gr4 hyperglycemia (Moreno Garcia et al., 2011; Von Hoff et al., 2011).

### BKM120 (Novartis)

BKM120 is an oral pyrimidine-derived pan-PI3K inhibitor with potent activity at nanomolar concentrations against all class I PI3K isoform while didn’t show any activity against other classes of PI3K as well as mTOR. *In vitro* preclinical models showed that BKM120 has a strong anti-proliferative activity in more than 400 cancer cell lines. The antitumor effects of BKM120 was also described in several xenograft models of lung cancer (Fruman and Rommel, 2011) and metastatic HER-2+ breast cancer (Nanni et al., 2012). Clinical data indicate that it is unlikely that BKM120 will achieve exposures sufficient to significantly engage the off-target activity at tolerated doses and schedules, however careful dose range selection is required to ensure specific targeting of PI3K signaling pathway (Brachmann et al., 2012). Given its ability to penetrate the blood-brain barrier, BKM120 may represent an attractive option for the treatment of glioblastoma multiforme (GBM), the most common and aggressive malignant primary brain tumor (Koul et al., 2012). The first-in-human phase I dose-escalation study investigated the MTD, safety, preliminary activity, and pharmacodynamics of BKM120 (Bendell et al., 2012). The study reports that BKM120 was well tolerated with a dose-dependent safety profile and it describes related side effects, such as hyperglycemia, rash, nausea, fatigue, and mood alterations. In particular hyperglycemia is consistent with inhibition of PI3K signaling and has been observed with other PI3K/mTOR/Akt pathway inhibitors. Disturbance of glucose homeostasis, as evidenced by hyperglycemia, was more common at higher doses and may be attributed to BKM120 inhibition of p110. Pharmacodynamics data demonstrate a dose-related inhibition of the PI3K signaling with significant decrease in pS6 phosphorylation and decreased [18F]fluorodeoxyglucose uptake. In another study, 77 patients with CRC, breast, lung, and endometrial cancers received oral BKM120 in monotherapy once daily (Call et al., 2010). PR were observed in two patients, a triple negative breast cancer with *KRAS* and *p53* mutation and a ER+/HER− metastatic breast cancer carrying *PIK3CA* mutations. At the same time, 58% of patients showed stable disease (SD) response.

### BAY 80-6946 (Bayer Healthcare)

BAY 80-6946 is a potent pan-class I PI3K inhibitor with IC50 at sub-nanomolar concentration against PI3Kα (0.5 nM), PI3Kβ (3.7 nM), PI3Kδ (0.7 nM), and PI3Kγ (6.4 nM) while it is inactive against around 240 protein/lipid kinases and RTKs. BAY 80-6946 has shown antitumor activity against a panel of 140 tumor cell lines with an IC50 of 1–100 nM in about 60 tumor cell lines. BAY 80-6946 displayed a strong activity of the PI3K signaling pathway inhibiting AKT (Thr308 and Ser473) as well as PRAS40, 4EBP1, and FOXOs phosphorylation in tumor cells carrying *PIK3CA* activating mutations. BAY 80-6946 has been demonstrated to induce apoptosis in a subset of *PIK3CA* mutant tumors at concentrations lower that 100 nM in preclinical studies. The pharmacokinetics, pharmacodynamics, and MTD of BAY 80-6946 have been determined in a phase I escalation multicenter study in patients with advanced solid tumors. Unlike other PI3K inhibitors, BAY 80-6946 is administered intravenously as 1-h infusion once weekly for 3 weeks every month. Data deriving from phase I study revealed a MTD at 0.8 mg/kg and several side effects including hyperglycemia, fatigue, nausea, alopecia, diarrhea, mucositis, dysgeusia, and grade 2/3 anemia. Moreover a phase I study assessed safety, pharmacokinetics, and clinical benefit in patients with advanced solid tumors, including breast, endometrial, gastric cancer.

### NVP-BEZ235 (Novartis)

NVP-BEZ235 is a reversible, orally available, and selective inhibitor of PI3K and TORC1/2. Several preclinical studies have already demonstrate its efficacy in a variety of solid tumors such as melanomas (Roper et al., 2011), breast (Brunner-Kubath et al., 2011), CRCs (Manara et al., 2010; Roper et al., 2011), and sarcomas (Manara et al., 2010). This compound suppresses cell proliferation, induces G1 cell cycle arrest and promotes autophagy by inhibiting the activity of AKT, S6K, S6, and 4EBP1 target proteins (Serra et al., 2008; Cerniglia et al., 2012). In addition, data on glioma xenograft demonstrate a reduction of the expression of the vascular endothelial growth factor (VEGF) on tumor vasculature, thus suggesting an anti-angiogenetic effect for NVP-BEZ235 (Liu et al., 2009b). Recently, NVP-BEZ235 also emerged as inhibitor of ATM and DNA-PK at low concentration (100 nM). In this context, treatment with NVP-BEZ235 may have significant radio sensitizing effects with important implications in the rational design of clinical trials (Mukherjee et al., 2012). At present, this molecule is under evaluation in phase I/II clinical trials in patients with advanced solid malignancies, including GBM (Salkeni et al., 2012), breast, renal cell carcinoma (RCC), castration-resistant prostate cancer (CRPC), endometrial carcinoma, and pancreatic neuroendocrine tumors (see text footnote 2), alone or in combination with other drugs such as Paclitaxel, Trastuzumab, Everolimus, and MEK162. A 3 + 3 dose-escalation schedule with NVP-BEZ235 revealed tolerability at 600 mg BID dose and preliminary signs of clinical and pharmacodynamic activity (Arkenau et al., 2012). In a phase IB dose-escalation study, NVP-BEZ235 was administered in combination with Trastuzumab in HER-2+ metastatic breast cancer (15 pts) with altered PI3K/PTEN status, showing an acceptable safety profile and PR or SD in one and four patients respectively (Krop et al., 2012). NVP-BEZ235 was also tested as a single agent or Trastuzumab-combined with a novel formulation based on a solid dispersion system (SDS) sachet (Peyton et al., 2011). The MTD for the new formulated NVP-BEZ235 was determined as 1600 mg/day, dose chosen for the ongoing phase II clinical trials. After treatment, in 28 patients with advanced solid tumors, a stable response has been evidence in a 40% of cases (Wen et al., 2012). Overall, these studies display that NVP-BEZ235 is generally well tolerated and the most common side effects include nausea, diarrhea, AST/ALT elevation, and headache. DLT include fatigue, asthenia, Gr3 thrombocytopenia, and Gr3 mucositis (Peyton et al., 2011; Arkenau et al., 2012; Wen et al., 2012).

## PI3K Signaling Inhibition in Hematological Malignancies

Although the majority of PI3K inhibitors are under development for the treatment of solid tumors, hematological malignancies also represent a therapeutic area of interest, especially for isoform-selective PI3K inhibitors. Constitutive activation of class I PI3K isoform has been identified in high percentage of acute and chronic leukemia. In addition, in some cases leukemias cell display overexpression of PI3Kα, PI3Kβ, and PI3Kγ. Conversely, the expression of PI3Kδ has been found up-regulated in acute myelogenous leukemia (AML) and in a subset of promyelocytic leukemia (APL). AML comprises a heterogeneous group of tumors characterized by uncontrolled proliferation of hematopoietic precursors thus leading to accumulation of blast cells in the bone marrow. These blasts are blocked in their differentiation program at different stage of maturation. This blast accumulation causes a progressive failure in the hematopoiesis process, which in turn leads to anemia, neutropenia, and thrombocytopenia (Smith et al., 2004). The standard therapeutic approach for the treatment of AML is based on high-dose chemotherapy, nonetheless the prognosis of AML remains poor with a 5-year survival rate in a 15–30% of patients. A recent evidence demonstrated that up-regulation of the PI3K/AKT/mTOR axis is a common feature in AML. Consequently, several pan-PI3K and dual PI3K/mTOR inhibitors, in particular BKM120 and BEZ235, are undergoing phase I clinical development to assess safety, dose, and preliminary efficacy in patients with advanced leukemias, relapsed or refractory acute lymphoblastic and myelocytic leukemia (see text footnote 2).

Several studies demonstrated that treatment with the PI3Kδ selective inhibitor, EC87114, inhibits AML cell proliferation without affecting the proliferation of normal hematopoietic progenitor cells (Sujobert et al., 2005; Billottet et al., 2006). In this context, PI3Kδ inhibitors represent a promising therapeutic treatment for AML without producing undesirable side effects that are conversely expected for other pan-PI3K inhibitors (Di Nicolantonio et al., 2010). Additionally, since PI3Kδ is expressed in leukocytes and plays a key role in B-cell signaling (Jou et al., 2002; Bilancio et al., 2006), it represents an interesting target in B-cells malignancies. Mice with deleted or kinase dead PI3Kδ exhibit B-cell defects, such as lack of B1 lymphocytes, decreased number of mature B-cell and impaired antibody production. The absence of PI3Kδ in B-cell leads also to a reduction in AKT phosphorylation and decreased PtdIns(3,4,5)P_3_ levels (Okkenhaug and Vanhaesebroeck, 2003). All these data support the use of PI3Kδ inhibitors in B-cell malignancies including the chronic lymphocytic leukemia (CLL).

Given the increasing interest in inhibiting PI3Kδ for the treatment of hematopoietic malignancies, several inhibitors are currently under evaluation in preclinical and in clinical trials. Presently, the most promising PI3Kδ inhibitor is represent by CAL-101 (Calistoga Pharmaceuticals/Gilead Sciences), an orally available selective inhibitor with an IC50 of 2.5 nM for PI3Kδ and 820, 565, 89 nM for PI3Kα, PI3Kβ, PI3Kγ respectively (Lannutti et al., 2011). At the present, CAL-101 is undergoing in preclinical and clinical development in a variety of lymphoid malignancies thanks to its high selectivity. Phase I studies, on patients with relapsed or refractory hematologic malignancies, including non-Hodgkin lymphoma (NHL), CLL, and AML, revealed a very favorable toxicity profile and pharmacokinetics during a week of CAL-101 oral administration (Fruman and Rommel, 2011). The predominant toxicity, caused by high serum transaminases, was observed in 21% of patients at doses of 200 and 350 mg. However, this side effect resulted to be reversible disappearing with a temporary discontinuation of the drug (Fruman and Rommel, 2011). In this study, 57 patients were treated with different dosages, corresponding to 50, 100, 200, and 350 mg. Clinical responses were seen at all dose levels resulting in 50% of reduction in lymphadenopathy and around 6 months of stable disease. In a successive phase I clinical trial CAL-101 was administered orally one or two times per day for a cycle of 28 day in 54 patients with CLL. After treatment with CAL-101, 26% of patients achieved a PR with 80% of patients showing reduced lymphadenopathy by ≥50% (Di Nicolantonio et al., 2010; Fruman and Rommel, 2011).

## Relevance for Selective Inhibition of Class II PI3Ks

Pharmacological inhibitors selectively targeting class II PI3Ks have not been described yet. In particular, *in vitro* PI3K-C2α is refractory to inhibition by LY294002 and Wortmannin (Virbasius et al., 1996; Domin et al., 1997), two very well-known PI3Ks inhibitors. However, emerging evidence suggests that class II PI3Ks may have crucial role in different types of tumor independently from class I PI3K activity.

### PI3K-C2α

RNAi-based silencing of PI3K-C2α in a large set of cancer cell lines showed that this enzyme is crucially required for cancer cell survival *in vitro* (Elis et al., 2008). A small but significantly difference in the DNA copy number and mRNA levels, was then reported *in vivo* in 19 hepatitis B-positive hepatocellular carcinoma compared with non-tumor tissues counterparts (Ng et al., 2009). The PIK3C2A gene was also found to be up-regulated in breast cancer stem-like cells characterized by increased tumorigenicity compared with the normal counterpart (Zhou et al., 2007). Moreover, it was reported that PI3K-C2α is directly down-modulated at a translational level by *miR-30e-3p* in DLD1 CRC cells (Schepeler et al., 2012). In particular *miR-30e-3p* levels are significantly decreased during early events of CRC carcinogenesis thus raising the possibility that PIK3C2A gene may be up-regulated in the initial steps of CRC onset. On the other hand, Yoshioka et al. (2012) showed that PI3K-C2α has an essential role during angiogenesis process and in vascular barrier function. After subcutaneous injection of Lewis lung carcinoma (LLC) or B16-BL6 melanoma tumors, Pik3c2a endothelial-restricted knock-out mice had reduced tumor volumes/weights compared to control, suggesting that the *in vivo* pro-angiogenetic function of PI3K-C2α is required for tumor growth and maintenance. Further studies are needed to better understand the role PI3K-C2α in tumor angiogenesis, however designing of specific inhibitors targeting PI3K-C2α could represent a promising new anti-angiogenetic approach to arrest tumor growth.

### PI3K-C2β

An increasing number of studies described the involvement of the gene PIK3C2B, encoding for PI3K-C2β, in cancer. Amplification of *PIK3C2B* gene at 1q32 was reported in 6 out of 103 GBM tumors and in 4 of these cases amplification correlates with *PI3KC2B* mRNA over-expression (Knobbe and Reifenberger, 2003). Amplification of chromosomal region 1q32.1 (*PIK3C2B\MDM4*) was also reported in around 8% of GBM tumor samples (Rao et al., 2010) and in whole genome amplification analysis (Nobusawa et al., 2010). On the other hand increased PI3K-C2β protein levels significantly correlates with resistance to Erlotinib in GBM thus suggesting that targeting of PI3K-C2β in resistant GBM may represent a new therapeutic approach (Low et al., 2008).

To further support the hypothesis the role of PI3K-C2β in drug resistance, a siRNA-based study reported that in a panel of kinases, down regulation PIK3C2B is one of the Tamoxifen sensitizing target in breast cancer cells (Iorns et al., 2009). Furthermore, over-expression of PI3K-C2β significantly inhibited cisplatin-induced apoptosis and cleavage of caspase-3 in esophageal squamous cell carcinoma (Liu et al., 2011). Altogether these data suggest that PI3K-C2β may have a role in promoting resistance to chemotherapeutic drugs and that interference with PI3K-C2β activity might be a rational possibility for treatment of cisplatin-resistant esophageal cancer patients. On the other hand, it has been recently showed that down regulation of PI3K-C2β may specifically confer resistance to leukemia cells to chemotherapy (thioguanine and mercaptopurine) (Diouf et al., 2011). Reduced levels of PI3K-C2β results in increased degradation of MSH2, a DNA mismatch repair enzyme involved in genomic integrity maintenance and drug resistance. Although it’s becoming increasingly clear the involvement of PI3K-C2β in chemotherapy resistance, further studies are needed to clarify when the targeting of this enzyme could promote drug sensitivity.

A recent study also reported that PIK3C2B gene single-nucleotide polymorphisms (SNP) is associated to cancer risk susceptibility (Koutros et al., 2010). The authors examined the association between several SNPs in PI3Ks genes (PIK3CD, PIK3C2A, PIK3R3, PIK3AP1, and PIK3C2B) and prostate cancer risk: among the five genes, only PIK3C2B showed a cluster of SNPs related to prostate cancer risk. PIK3C2B was also found to be significantly mutated in a recent whole-exome sequencing screening in NSCLC (Liu et al., 2012).

### PI3K-C2γ

At the present very little is known about the role of PI3K-C2γ in cancer. The chromosomal region 12p12, containing the human PIK3C2G gene, is found significantly amplified in a subset of ovarian cancer (Lambros et al., 2005) and in about 60% of pancreatic ductal adenocarcinoma (PDAC) (Harada et al., 2008). Recently bioinformatics analysis reported significative association between PIK3C2G mutations and GBM related signaling pathways (Dong et al., 2010). Another study also suggests a new mechanism through which Bcr-Abl induces abnormal homing of leukemia cells by reducing PI3K-C2γ expression (Yu et al., 2010). In this way, Bcr-Abl is not only responsible for class I PI3K activation in cell migration (Martelli et al., 2006) but also for PI3K-C2γ reduced expression (Yu et al., 2010).

Altogether these data suggest that class II PI3Ks may exert additional or complementary role to class I PI3Ks in promoting cancer, being involved in specific kind of tumors. The development of selective inhibitors targeting class II PI3Ks may thus represent a promising way of action, once the precise role of these enzymes in cancer will be elucidated.

## Future Perspectives

As PI3K inhibitors progress into trials focusing on their clinical efficacy, it is critical to identify genomic determinants of response and to stratify the patient population that will most likely benefit from the treatment (Weigelt and Downward, 2012).

Historically rapalogs were the first PI3K pathway inhibitors tested in clinical trials for cancer therapy and currently preclinical models and early clinical data suggested that *PIK3CA* mutations may predict sensitivity to treatment with PI3K/AKT/mTOR inhibitors (Engelman et al., 2008; Ihle et al., 2009). In particular, it has been shown that patients with advanced malignancies carrying *PIK3CA* pathway aberrations, showed high percentage of response to rapalogs and/or PI3K pan-inhibitor (Janku et al., 2011b; Moroney et al., 2011). Moreover, PI3K pathway aberrations due to *PIK3CA* mutations or PTEN loss correlate with increase response to Doxorubicin, Bevacizumab, and Temsirolimus in patients with advanced gynecologic and breast malignancies (Moroney et al., 2011). Likewise, ovarian cancers with coexisting *PIK3CA* and *MAPK* pathway mutations are sensitive to PI3K inhibition, whereas CRC with the same repertoire of mutations are resistant (Di Nicolantonio et al., 2010; Janku et al., 2011a, 2012). The identification of feedback loops leading to MAPK activation, upon PI3K inhibition, underscores the potential of a combined therapeutic approach with PI3K and MAPK inhibitors (Carracedo et al., 2008; Laplante and Sabatini, 2012). According to these findings, it has been proposed to incorporate predictive biomarkers during the clinical drug development process from phase I studies onward in order to enrich trials with patients more likely to respond to a given targeted therapy and to increase the chances of drug registration (Carden et al., 2010). For the guidance and prioritization of predictive biomarker candidates in early clinical trials, results derived from the study of preclinical models are of primary importance to develop accurate companion diagnostic tools.

## Conclusion

In summary, although available dataset showed that a high percentage of tumors harboring *PIK3CA* will likely benefit from inhibition of the PI3K pathway, a substantial proportion of patients with *PIK3CA* activating mutations may be *de novo* resistant to these agents. On the other hand, not all the patients with *PIK3CA* mutations are sensitive to PI3K inhibitors and not all the patients with wt *PIK3CA*/*PTEN* tumors are responsive. Moreover, while the majority of the studies have restricted the analysis on the *PIK3CA* and PTEN status, also occurring in other members of the pathway, such as mTOR activating mutations, or INPP4B loss of function, may play a role in the response to inhibitors. Therefore, the possibility to target PI3K signaling pathway in cancer requires deeper investigation, in order to identify additional biomarkers and to improve therapeutic strategies in the clinic.

## Conflict of Interest Statement

The authors declare that the research was conducted in the absence of any commercial or financial relationships that could be construed as a potential conflict of interest.

## Footnotes

^1^www.sanger.ac.uk/cosmic

^2^www.clinicaltrials.gov

## References

[B1] Antonescu C. R. (2011). The GIST paradigm: lessons for other kinase-driven cancers. J. Pathol. 223, 251–261 10.1002/path.2798 21125679 PMC4225081

[B2] Arcaro A. Khanzada U. K. Vanhaesebroeck B. Tetley T. D. Waterfield M. D. Seckl M. J. (2002). Two distinct phosphoinositide 3-kinases mediate polypeptide growth factor-stimulated PKB activation. EMBO J. 21, 5097–5108 10.1093/emboj/cdf512 12356726 PMC129034

[B3] Arcaro A. Zvelebil M. J. Wallasch C. Ullrich A. Waterfield M. D. Domin J. (2000). Class II phosphoinositide 3-kinases are downstream targets of activated polypeptide growth factor receptors. Mol. Cell. Biol. 20, 3817–3830 10.1128/MCB.20.11.3817-3830.2000 10805725 PMC85707

[B4] Arkenau H.-T. Jones S. F. Kurkjian C. Infante J. R. Pant S. Burris H. A. (2012). The PI3K/mTOR inhibitor BEZ235 given twice daily for the treatment of patients (pts) with advanced solid tumors. J. Clin. Oncol. 30, abstr. 3097. [ASCO Annual Meeting Proceedings].

[B5] Bendell J. C. Rodon J. Burris H. A. De Jonge M. Verweij J. Birle D. (2012). Phase I, dose-escalation study of BKM120, an oral pan-Class I PI3K inhibitor, in patients with advanced solid tumors. J. Clin. Oncol. 30, 282–290 10.1200/JCO.2011.36.1360 22162589

[B6] Bilancio A. Okkenhaug K. Camps M. Emery J. L. Ruckle T. Rommel C. (2006). Key role of the p110delta isoform of PI3K in B-cell antigen and IL-4 receptor signaling: comparative analysis of genetic and pharmacologic interference with p110delta function in B cells. Blood 107, 642–650 10.1182/blood-2005-07-3041 16179367

[B7] Billottet C. Grandage V. L. Gale R. E. Quattropani A. Rommel C. Vanhaesebroeck B. (2006). A selective inhibitor of the p110delta isoform of PI 3-kinase inhibits AML cell proliferation and survival and increases the cytotoxic effects of VP16. Oncogene 25, 6648–6659 10.1038/sj.onc.1209670 16702948

[B8] Brachmann S. M. Kleylein-Sohn J. Gaulis S. Kauffmann A. Blommers M. J. Kazic-Legueux M. (2012). Characterization of the mechanism of action of the pan class I PI3K inhibitor NVP-BKM120 across a broad range of concentrations. Mol. Cancer Ther. 11, 1747–1757 10.1158/1535-7163.MCT-11-1021 22653967

[B9] Brown R. A. Domin J. Arcaro A. Waterfield M. D. Shepherd P. R. (1999). Insulin activates the alpha isoform of class II phosphoinositide 3-kinase. J. Biol. Chem. 274, 14529–14532 10.1074/jbc.274.37.26272 10329640

[B10] Brunner-Kubath C. Shabbir W. Saferding V. Wagner R. Singer C. F. Valent P. (2011). The PI3 kinase/mTOR blocker NVP-BEZ235 overrides resistance against irreversible ErbB inhibitors in breast cancer cells. Breast Cancer Res. Treat. 129, 387–400 10.1007/s10549-010-1232-1 21046231

[B11] Call R. Grimsley M. Cadwallader L. Cialone L. Hill M. Hreish V. (2010). Insulin – carcinogen or mitogen? Preclinical and clinical evidence from prostate, breast, pancreatic, and colorectal cancer research. Postgrad. Med. 122, 158–165 10.3810/pgm.2010.05.2153 20463425

[B12] Cantley L. C. (2002). The phosphoinositide 3-kinase pathway. Science 296, 1655–1657 10.1126/science.296.5573.1655 12040186

[B13] Carden C. P. Sarker D. Postel-Vinay S. Yap T. A. Attard G. Banerji U. (2010). Can molecular biomarker-based patient selection in Phase I trials accelerate anticancer drug development? Drug Discov. Today 15, 88–97 10.1016/j.drudis.2009.11.006 19961955

[B14] Carracedo A. Ma L. Teruya-Feldstein J. Rojo F. Salmena L. Alimonti A. (2008). Inhibition of mTORC1 leads to MAPK pathway activation through a PI3K-dependent feedback loop in human cancer. J. Clin. Invest. 118, 3065–3074 18725988 10.1172/JCI34739 PMC2518073

[B15] Cerniglia G. J. Karar J. Tyagi S. Christofidou-Solomidou M. Rengan R. Koumenis C. (2012). Inhibition of autophagy as a strategy to augment radiosensitization by the dual phosphatidylinositol 3-kinase/mammalian target of rapamycin inhibitor NVP-BEZ235. Mol. Pharmacol. 82, 1230–1240 10.1124/mol.112.080408 22989521 PMC3502620

[B16] Di Nicolantonio F. Arena S. Tabernero J. Grosso S. Molinari F. MacArulla T. (2010). Deregulation of the PI3K and KRAS signaling pathways in human cancer cells determines their response to everolimus. J. Clin. Invest. 120, 2858–2866 10.1172/JCI37539 20664172 PMC2912177

[B17] Diouf B. Cheng Q. Krynetskaia N. F. Yang W. Cheok M. Pei D. (2011). Somatic deletions of genes regulating MSH2 protein stability cause DNA mismatch repair deficiency and drug resistance in human leukemia cells. Nat. Med. 17, 1298–1303 10.1038/nm.2430 21946537 PMC3192247

[B18] Domin J. Pages F. Volinia S. Rittenhouse S. E. Zvelebil M. J. Stein R. C. (1997). Cloning of a human phosphoinositide 3-kinase with a C2 domain that displays reduced sensitivity to the inhibitor wortmannin. Biochem. J. 326(Pt 1), 139–147 9337861 10.1042/bj3260139 PMC1218647

[B19] Dong H. Luo L. Hong S. Siu H. Xiao Y. Jin L. (2010). Integrated analysis of mutations, miRNA and mRNA expression in glioblastoma. BMC Syst. Biol. 4:163 10.1186/1752-0509-4-163 21114830 PMC3002314

[B20] Elis W. Triantafellow E. Wolters N. M. Sian K. R. Caponigro G. Borawski J. (2008). Down-regulation of class II phosphoinositide 3-kinase alpha expression below a critical threshold induces apoptotic cell death. Mol. Cancer Res. 6, 614–623 10.1158/1541-7786.MCR-07-0262 18403640

[B21] Engelman J. A. Chen L. Tan X. Crosby K. Guimaraes A. R. Upadhyay R. (2008). Effective use of PI3K and MEK inhibitors to treat mutant Kras G12D and PIK3CA H1047R murine lung cancers. Nat. Med. 14, 1351–1356 10.1038/nm.1890 19029981 PMC2683415

[B22] Falasca M. Hughes W. E. Dominguez V. Sala G. Fostira F. Fang M. Q. (2007). The role of phosphoinositide 3-kinase C2alpha in insulin signaling. J. Biol. Chem. 282, 28226–28236 10.1074/jbc.M704357200 17644513

[B23] Falasca M. Maffucci T. (2007). Role of class II phosphoinositide 3-kinase in cell signalling. Biochem. Soc. Trans. 35, 211–214 10.1042/BST0350229 17371240

[B24] Flaherty K. T. Puzanov I. Kim K. B. Ribas A. McArthur G. A. Sosman J. A. (2010). Inhibition of mutated, activated BRAF in metastatic melanoma. N. Engl. J. Med. 363, 809–819 10.1056/NEJMoa1002011 20818844 PMC3724529

[B25] Folkes A. J. Ahmadi K. Alderton W. K. Alix S. Baker S. J. Box G. (2008). The identification of 2-(1H-indazol-4-yl)-6-(4-methanesulfonyl-piperazin-1-ylmethyl)-4-morpholin-4-yl-t hieno[3,2-d]pyrimidine (GDC-0941) as a potent, selective, orally bioavailable inhibitor of class I PI3 kinase for the treatment of cancer. J. Med. Chem. 51, 5522–5532 10.1021/jm800295d 18754654

[B26] Fruman D. A. Rommel C. (2011). PI3Kdelta inhibitors in cancer: rationale and serendipity merge in the clinic. Cancer Discov. 1, 562–572 10.1158/2159-8290.CD-11-0249 22586681

[B27] Gaidarov I. Smith M. E. Domin J. Keen J. H. (2001). The class II phosphoinositide 3-kinase C2alpha is activated by clathrin and regulates clathrin-mediated membrane trafficking. Mol. Cell 7, 443–449 10.1016/S1097-2765(01)00191-5 11239472

[B28] Gewinner C. Wang Z. C. Richardson A. Teruya-Feldstein J. Etemadmoghadam D. Bowtell D. (2009). Evidence that inositol polyphosphate 4-phosphatase type II is a tumor suppressor that inhibits PI3K signaling. Cancer Cell 16, 115–125 10.1016/j.ccr.2009.06.006 19647222 PMC2957372

[B29] Harada T. Chelala C. Bhakta V. Chaplin T. Caulee K. Baril P. (2008). Genome-wide DNA copy number analysis in pancreatic cancer using high-density single nucleotide polymorphism arrays. Oncogene 27, 1951–1960 10.1038/sj.onc.1210832 17952125 PMC2492386

[B30] Hollander M. C. Blumenthal G. M. Dennis P. A. (2011). PTEN loss in the continuum of common cancers, rare syndromes and mouse models. Nat. Rev. Cancer 11, 289–301 10.1038/nri2959 21430697 PMC6946181

[B31] Ihle N. T. Lemos R. Jr. Wipf P. Yacoub A. Mitchell C. Siwak D. (2009). Mutations in the phosphatidylinositol-3-kinase pathway predict for antitumor activity of the inhibitor PX-866 whereas oncogenic Ras is a dominant predictor for resistance. Cancer Res. 69, 143–150 10.1158/0008-5472.CAN-07-6656 19117997 PMC2613546

[B32] Iorns E. Lord C. J. Ashworth A. (2009). Parallel RNAi and compound screens identify the PDK1 pathway as a target for tamoxifen sensitization. Biochem. J. 417, 361–370 10.1042/BJ20081682 18976239

[B33] Janku F. Lee J. J. Tsimberidou A. M. Hong D. S. Naing A. Falchook G. S. (2011a). PIK3CA mutations frequently coexist with RAS and BRAF mutations in patients with advanced cancers. PLoS ONE 6:e22769 10.1371/journal.pone.0022769 21829508 PMC3146490

[B34] Janku F. Tsimberidou A. M. Garrido-Laguna I. Wang X. Luthra R. Hong D. S. (2011b). PIK3CA mutations in patients with advanced cancers treated with PI3K/AKT/mTOR axis inhibitors. Mol. Cancer Ther. 10, 558–565 10.1158/1535-7163.MCT-10-0994 21216929 PMC3072168

[B35] Janku F. Wheler J. J. Naing A. Stepanek V. M. Falchook G. S. Fu S. (2012). PIK3CA mutations in advanced cancers: characteristics and outcomes. Oncotarget 3, 1566–1575 23248156 10.18632/oncotarget.716 PMC3681495

[B36] Janne P. A. Gray N. Settleman J. (2009). Factors underlying sensitivity of cancers to small-molecule kinase inhibitors. Nat. Rev. Drug Discov. 8, 709–723 10.1038/nrd2871 19629074

[B37] Jou S. T. Carpino N. Takahashi Y. Piekorz R. Chao J. R. Carpino N. (2002). Essential, nonredundant role for the phosphoinositide 3-kinase p110delta in signaling by the B-cell receptor complex. Mol. Cell. Biol. 22, 8580–8591 10.1128/MCB.22.24.8580-8591.2002 12446777 PMC139888

[B38] Kim J. S. Yun H. S. Um H. D. Park J. K. Lee K. H. Kang C. M. (2012). Identification of inositol polyphosphate 4-phosphatase type II as a novel tumor resistance biomarker in human laryngeal cancer HEp-2 cells. Cancer Biol. Ther. 13, 1307–1318 10.4161/cbt.21788 22895072 PMC3493439

[B39] Knobbe C. B. Reifenberger G. (2003). Genetic alterations and aberrant expression of genes related to the phosphatidyl-inositol-3’-kinase/protein kinase B (Akt) signal transduction pathway in glioblastomas. Brain Pathol. 13, 507–518 10.1111/j.1750-3639.2003.tb00481.x 14655756 PMC8095764

[B40] Kok K. Geering B. Vanhaesebroeck B. (2009). Regulation of phosphoinositide 3-kinase expression in health and disease. Trends Biochem. Sci. 34, 115–127 10.1016/j.tibs.2009.01.003 19299143

[B41] Koul D. Fu J. Shen R. Lafortune T. A. Wang S. Tiao N. (2012). Antitumor activity of NVP-BKM120 – a selective pan class I PI3 kinase inhibitor showed differential forms of cell death based on p53 status of glioma cells. Clin. Cancer Res. 18, 184–195 10.1158/1078-0432.CCR-11-1558 22065080 PMC3785365

[B42] Koutros S. Schumacher F. R. Hayes R. B. Ma J. Huang W. Y. Albanes D. (2010). Pooled analysis of phosphatidylinositol 3-kinase pathway variants and risk of prostate cancer. Cancer Res. 70, 2389–2396 10.1158/0008-5472.CAN-09-3575 20197460 PMC2840184

[B43] Krop I. E. Saura C. Ahnert J. R. Becerra C. Britten C. D. Isakoff S. J. (2012). A phase I/IB dose-escalation study of BEZ235 in combination with trastuzumab in patients with PI3-kinase or PTEN altered HER2+ metastatic breast cancer. J. Clin. Oncol. 30, abstr. 508. [ASCO Annual Meeting Proceedings]. 10.1200/JCO.2011.40.5902

[B44] Ktori C. Shepherd P. R. O’Rourke L. (2003). TNF-alpha and leptin activate the alpha-isoform of class II phosphoinositide 3-kinase. Biochem. Biophys. Res. Commun. 306, 139–143 10.1016/S0006-291X(03)00933-1 12788079

[B45] Lambros M. B. Fiegler H. Jones A. Gorman P. Roylance R. R. Carter N. P. (2005). Analysis of ovarian cancer cell lines using array-based comparative genomic hybridization. J. Pathol. 205, 29–40 10.1002/path.1681 15586366

[B46] Lannutti B. J. Meadows S. A. Herman S. E. Kashishian A. Steiner B. Johnson A. J. (2011). CAL-101, a p110delta selective phosphatidylinositol-3-kinase inhibitor for the treatment of B-cell malignancies, inhibits PI3K signaling and cellular viability. Blood 117, 591–594 10.1182/blood-2010-03-275305 20959606 PMC3694505

[B47] Laplante M. Sabatini D. M. (2012). mTOR signaling in growth control and disease. Cell 149, 274–293 10.1016/j.cell.2012.03.017 22500797 PMC3331679

[B48] Liu P. Cheng H. Roberts T. M. Zhao J. J. (2009a). Targeting the phosphoinositide 3-kinase pathway in cancer. Nat. Rev. Drug Discov. 8, 627–644 10.1038/nrd2926 19644473 PMC3142564

[B49] Liu T. J. Koul D. Lafortune T. Tiao N. Shen R. J. Maira S. M. (2009b). NVP-BEZ235, a novel dual phosphatidylinositol 3-kinase/mammalian target of rapamycin inhibitor, elicits multifaceted antitumor activities in human gliomas. Mol. Cancer Ther. 8, 2204–2210 10.1158/1535-7163.TARG-09-C106 19671762 PMC2752877

[B50] Liu P. Morrison C. Wang L. Xiong D. Vedell P. Cui P. (2012). Identification of somatic mutations in non-small cell lung carcinomas using whole-exome sequencing. Carcinogenesis 33, 1270–1276 10.1093/carcin/bgs098 22510280 PMC3499051

[B51] Liu Z. Sun C. Zhang Y. Ji Z. Yang G. (2011). Phosphatidylinositol 3-kinase-C2beta inhibits cisplatin-mediated apoptosis via the Akt pathway in oesophageal squamous cell carcinoma. J. Int. Med. Res. 39, 1319–1332 21986133 10.1177/147323001103900419

[B52] Low S. Vougioukas V. I. Hielscher T. Schmidt U. Unterberg A. Halatsch M. E. (2008). Pathogenetic pathways leading to glioblastoma multiforme: association between gene expressions and resistance to erlotinib. Anticancer Res. 28, 3729–3732 19189657

[B53] Maffucci T. Cooke F. T. Foster F. M. Traer C. J. Fry M. J. Falasca M. (2005). Class II phosphoinositide 3-kinase defines a novel signaling pathway in cell migration. J. Cell Biol. 169, 789–799 10.1083/jcb.200408005 15928202 PMC2171608

[B54] Manara M. C. Nicoletti G. Zambelli D. Ventura S. Guerzoni C. Landuzzi L. (2010). NVP-BEZ235 as a new therapeutic option for sarcomas. Clin. Cancer Res. 16, 530–540 10.1158/1078-0432.CCR-09-0816 20068094

[B55] Martelli A. M. Nyakern M. Tabellini G. Bortul R. Tazzari P. L. Evangelisti C. (2006). Phosphoinositide 3-kinase/Akt signaling pathway and its therapeutical implications for human acute myeloid leukemia. Leukemia 20, 911–928 10.1038/sj.leu.2404245 16642045

[B56] Martini M. Vecchione L. Siena S. Tejpar S. Bardelli A. (2012). Targeted therapies: how personal should we go? Nat. Rev. Clin. Oncol. 9, 87–97 10.1038/nrclinonc.2011.164 22083042

[B57] Miled N. Yan Y. Hon W. C. Perisic O. Zvelebil M. Inbar Y. (2007). Mechanism of two classes of cancer mutations in the phosphoinositide 3-kinase catalytic subunit. Science 317, 239–242 10.1126/science.1135394 17626883

[B58] Moreno Garcia V. Baird R. D. Shah K. J. Basu B. Tunariu N. Blanco M. (2011). A phase I study evaluating GDC-0941, an oral phosphoinositide-3 kinase (PI3K) inhibitor, in patients with advanced solid tumors or multiple myeloma. J. Clin. Oncol. 29, 3021 [ASCO Annual Meeting Proceedings].

[B59] Moroney J. W. Schlumbrecht M. P. Helgason T. Coleman R. L. Moulder S. Naing A. (2011). A phase I trial of liposomal doxorubicin, bevacizumab, and temsirolimus in patients with advanced gynecologic and breast malignancies. Clin. Cancer Res. 17, 6840–6846 10.1158/1078-0432.CCR-11-0666 21890452 PMC3207033

[B60] Mukherjee B. Tomimatsu N. Amancherla K. Camacho C. V. Pichamoorthy N. Burma S. (2012). The dual PI3K/mTOR inhibitor NVP-BEZ235 is a potent inhibitor of ATM- and DNA-PKCs-mediated DNA damage responses. Neoplasia 14, 34–43 22355272 10.1593/neo.111512 PMC3281940

[B61] Nanni P. Nicoletti G. Palladini A. Croci S. Murgo A. Ianzano M. L. (2012). Multiorgan metastasis of human HER-2+ breast cancer in Rag2-/-;Il2rg-/- mice and treatment with PI3K inhibitor. PLoS ONE 7:e39626 10.1371/journal.pone.0039626 22737248 PMC3380859

[B62] Ng S. K. Neo S. Y. Yap Y. W. Karuturi R. K. Loh E. S. Liau K. H. (2009). Ablation of phosphoinositide-3-kinase class II alpha suppresses hepatoma cell proliferation. Biochem. Biophys. Res. Commun. 387, 310–315 10.1016/j.bbrc.2009.07.013 19591801

[B63] Nobusawa S. Lachuer J. Wierinckx A. Kim Y. H. Huang J. Legras C. (2010). Intratumoral patterns of genomic imbalance in glioblastomas. Brain Pathol. 20, 936–944 20406234 10.1111/j.1750-3639.2010.00395.x PMC8094667

[B64] Okkenhaug K. Vanhaesebroeck B. (2003). PI3K-signalling in B- and T-cells: insights from gene-targeted mice. Biochem. Soc. Trans. 31, 270–274 10.1042/BST0310270 12546700

[B65] Pallis A. G. Fennell D. A. Szutowicz E. Leighl N. B. Greillier L. Dziadziuszko R. (2011). Biomarkers of clinical benefit for anti-epidermal growth factor receptor agents in patients with non-small-cell lung cancer. Br. J. Cancer 105, 1–8 10.1038/bjc.2011.416 21654681 PMC3137421

[B66] Peyton J. D. Rodon Ahnert J. Burris H. Britten C. Chen L. C. Tabernero J. (2011). A dose-escalation study with the novel formulation of the oral pan-class I PI3K inhibitor BEZ235, solid dispersion system (SDS) sachet, in patients with advanced solid tumors. J. Clin. Oncol. 29, abstr. 3066. [ASCO Annual Meeting Proceedings].

[B67] Rao S. K. Edwards J. Joshi A. D. Siu I. M. Riggins G. J. (2010). A survey of glioblastoma genomic amplifications and deletions. J. Neurooncol. 96, 169–179 10.1007/s11060-009-9959-4 19609742

[B68] Roper J. Richardson M. P. Wang W. V. Richard L. G. Chen W. Coffee E. M. (2011). The dual PI3K/mTOR inhibitor NVP-BEZ235 induces tumor regression in a genetically engineered mouse model of PIK3CA wild-type colorectal cancer. PLoS ONE 6:e25132 10.1371/journal.pone.0025132 21966435 PMC3180374

[B69] Salkeni M. A. Beg M. S. Olowokure O. O. Fathallah H. Thomas H. Mercer C. A. (2012). A dose escalation, single arm, phase Ib/II combination study of BEZ235 with everolimus to determine the safety, pharmacodynamics, and pharmacokinetics in subjects with advanced solid malignancies including glioblastoma multiforme. J. Clin. Oncol. 30, TPS3116 [ASCO Annual Meeting Proceedings].

[B70] Samuels Y. Wang Z. Bardelli A. Silliman N. Ptak J. Szabo S. (2004). High frequency of mutations of the PIK3CA gene in human cancers. Science 304, 554 10.1126/science.1096502 15016963

[B71] Schepeler T. Holm A. Halvey P. Nordentoft I. Lamy P. Riising E. M. (2012). Attenuation of the beta-catenin/TCF4 complex in colorectal cancer cells induces several growth-suppressive microRNAs that target cancer promoting genes. Oncogene 31, 2750–2760 10.1038/onc.2011.453 21963845

[B72] Serra V. Markman B. Scaltriti M. Eichhorn P. J. Valero V. Guzman M. (2008). NVP-BEZ235, a dual PI3K/mTOR inhibitor, prevents PI3K signaling and inhibits the growth of cancer cells with activating PI3K mutations. Cancer Res. 68, 8022–8030 10.1158/0008-5472.CAN-08-1385 18829560

[B73] Sharma S. V. Settleman J. (2007). Oncogene addiction: setting the stage for molecularly targeted cancer therapy. Genes Dev. 21, 3214–3231 10.1101/gad.1609907 18079171

[B74] Smith M. Barnett M. Bassan R. Gatta G. Tondini C. Kern W. (2004). Adult acute myeloid leukaemia. Crit. Rev. Oncol. Hematol. 50, 197–222 10.1016/j.critrevonc.2003.11.002 15182826

[B75] Steelman L. S. Navolanic P. M. Sokolosky M. L. Taylor J. R. Lehmann B. D. Chappell W. H. (2008). Suppression of PTEN function increases breast cancer chemotherapeutic drug resistance while conferring sensitivity to mTOR inhibitors. Oncogene 27, 4086–4095 10.1038/onc.2008.49 18332865 PMC3836277

[B76] Stern H. M. (2012). Improving treatment of HER2-positive cancers: opportunities and challenges. Sci. Transl. Med. 4, 127rv2 10.1126/scitranslmed.3001539 22461643

[B77] Sujobert P. Bardet V. Cornillet-Lefebvre P. Hayflick J. S. Prie N. Verdier F. (2005). Essential role for the p110delta isoform in phosphoinositide 3-kinase activation and cell proliferation in acute myeloid leukemia. Blood 106, 1063–1066 10.1182/blood-2004-08-3225 15840695

[B78] Turner S. J. Domin J. Waterfield M. D. Ward S. G. Westwick J. (1998). The CC chemokine monocyte chemotactic peptide-1 activates both the class I p85/p110 phosphatidylinositol 3-kinase and the class II PI3K-C2alpha. J. Biol. Chem. 273, 25987–25995 10.1074/jbc.273.40.25987 9748276

[B79] Vadas O. Burke J. E. Zhang X. Berndt A. Williams R. L. (2011). Structural basis for activation and inhibition of class I phosphoinositide 3-kinases. Sci. Signal. 4, re2 10.1126/scisignal.2002165 22009150

[B80] Vanhaesebroeck B. Guillermet-Guibert J. Graupera M. Bilanges B. (2010). The emerging mechanisms of isoform-specific PI3K signalling. Nat. Rev. Mol. Cell Biol. 11, 329–341 10.1038/nrm2882 20379207

[B81] Virbasius J. V. Guilherme A. Czech M. P. (1996). Mouse p170 is a novel phosphatidylinositol 3-kinase containing a C2 domain. J. Biol. Chem. 271, 13304–13307 10.1074/jbc.271.23.13304 8663140

[B82] Vogelstein B. Kinzler K. W. (2004). Cancer genes and the pathways they control. Nat. Med. 10, 789–799 10.1038/nm1087 15286780

[B83] Von Hoff D. D. Lorusso P. Demetri G. D. Weiss G. J. Shapiro G. Ramanathan R. K. (2011). A phase I dose-escalation study to evaluate GDC-0941, a pan-PI3K inhibitor, administered QD or BID in patients with advanced or metastatic solid tumors. J. Clin. Oncol. 29, 3052 [ASCO Annual Meeting Proceedings]. 10.1200/JCO.2011.36.5742

[B84] Wagner A. J. Von Hoff D. H. Lorusso P. M. Tibes R. Mazina K. E. Ware J. A. (2009). A first-in-human phase I study to evaluate the pan-PI3K inhibitor GDC-0941 administered QD or BID in patients with advanced solid tumors. J. Clin. Oncol. 27, abstr. 3501. [ASCO Annual Meeting Proceedings]. 10.1200/JCO.2008.18.5918

[B85] Wallin J. J. Guan J. Prior W. W. Lee L. B. Berry L. Belmont L. D. (2012). GDC-0941, a novel class I selective PI3K inhibitor, enhances the efficacy of docetaxel in human breast cancer models by increasing cell death in vitro and in vivo. Clin. Cancer Res. 18, 3901–3911 10.1158/1078-0432.CCR-11-2088 22586300

[B86] Weigelt B. Downward J. (2012). Genomic determinants of PI3K pathway inhibitor response in cancer. Front. Oncol. 2:109 10.3389/fonc.2012.00109 22970424 PMC3431500

[B87] Weinstein I. B. (2000). Disorders in cell circuitry during multistage carcinogenesis: the role of homeostasis. Carcinogenesis 21, 857–864 10.1093/carcin/21.5.857 10783304

[B88] Weinstein I. B. (2002). Cancer. Addiction to oncogenes – the Achilles heal of cancer. Science 297, 63–64 10.1126/science.1073096 12098689

[B89] Wen P. Y. Lee E. Q. Reardon D. A. Ligon K. L. Alfred Yung W. K. (2012). Current clinical development of PI3K pathway inhibitors in glioblastoma. Neuro-oncology 14, 819–829 10.1093/neuonc/nos117 22619466 PMC3379803

[B90] Yao E. Zhou W. Lee-Hoeflich S. T. Truong T. Haverty P. M. Eastham-Anderson J. (2009). Suppression of HER2/HER3-mediated growth of breast cancer cells with combinations of GDC-0941 PI3K inhibitor, trastuzumab, and pertuzumab. Clin. Cancer Res. 15, 4147–4156 10.1158/1078-0432.CCR-08-2814 19509167

[B91] Yoshioka K. Yoshida K. Cui H. Wakayama T. Takuwa N. Okamoto Y. (2012). Endothelial PI3K-C2alpha, a class II PI3K, has an essential role in angiogenesis and vascular barrier function. Nat. Med. 18, 1560–1569 10.1038/nm.2928 22983395

[B92] Yu W. Sun X. Tang H. Tao Y. Dai Z. (2010). Inhibition of class II phosphoinositide 3-kinase gamma expression by p185(Bcr-Abl) contributes to impaired chemotaxis and aberrant homing of leukemic cells. Leuk. Lymphoma 51, 1098–1107 10.3109/10428191003754624 20536348 PMC2885034

[B93] Zheng J. Zou X. Yao J. (2012). The antitumor effect of GDC-0941 alone and in combination with rapamycin in breast cancer cells. Chemotherapy 58, 273–281 10.1159/000341812 23006739

[B94] Zhou J. Wulfkuhle J. Zhang H. Gu P. Yang Y. Deng J. (2007). Activation of the PTEN/mTOR/STAT3 pathway in breast cancer stem-like cells is required for viability and maintenance. Proc. Natl. Acad. Sci. U.S.A. 104, 16158–16163 10.1073/pnas.0702409104 17911267 PMC2042178

[B95] Zou Z. Q. Zhang L. N. Wang F. Bellenger J. Shen Y. Z. Zhang X. H. (2012). The novel dual PI3K/mTOR inhibitor GDC-0941 synergizes with the MEK inhibitor U0126 in non-small cell lung cancer cells. Mol. Med. Rep. 5, 503–508 22101421 10.3892/mmr.2011.682

